# Unveiling the nephrotoxic profile of BCR‐ABL tyrosine kinase inhibitors: A real‐world experience in Africa

**DOI:** 10.1002/jha2.988

**Published:** 2024-07-31

**Authors:** Zekarias Seifu Ayalew, Gebeyehu Tessema Azibte, Fisihatsion Tadesse, Biruk Abate Legesse, Zerubabel Getahun Kiflu, Mahlet Tsige Weldeamanuel, Kibrekidusan Aynekulu Tsige, Bereket Abraha Molla, Addisu Melkie Ejigu

**Affiliations:** ^1^ Department of Internal Medicine Addis Ababa University Addis Ababa Ethiopia; ^2^ Department of Internal Medicine, Division of Hematology Addis Ababa University Addis Ababa Ethiopia; ^3^ Department of Clinical Oncology Addis Ababa University Addis Ababa Ethiopia; ^4^ Department of Internal Medicine, Division of Nephrology Addis Ababa University Addis Ababa Ethiopia

**Keywords:** BCR‐ABL, CML, renal toxicities, tyrosine kinase inhibitors

## Abstract

**Introduction:**

The efficacy of BCR‐ABL tyrosine kinase inhibitors (TKIs) in treating chronic myelogenous leukemia and other malignancies is well‐documented. However, concerns about potential nephrotoxicity have raised questions. This study, conducted at Tikur Anbesa Specialized Hospital (TASH) in Addis Ababa, Ethiopia, aimed to investigate the association between TKIs and renal toxicities.

**Methods:**

A hospital‐based cross‐sectional design was used to enroll 260 TASH patients actively receiving BCR‐ABL TKIs. Demographic information, diagnoses, treatment details, and laboratory test results were collected for each participant's Electronic Medical Record. The primary goal was to assess adverse renal events, a combination of events of a decrease in estimated glomerular filtration rate (eGFR) exceeding 30% from baseline, significant proteinuria, and a diagnosis of acute kidney injury (AKI) or chronic kidney disease (CKD). A logistic regression model was used to analyze the data and identify factors associated with developing adverse renal events.

**Results:**

Our analysis revealed a statistically significant decrease in eGFR following treatment with TKIs. However, the observed rate of adverse renal events (13.1%) was lower than reported in some previous studies. Factors significantly associated with adverse renal events included longer TKI duration, male sex (protective), hypertension, HIV infection, and achieving complete molecular remission and/or a complete hematologic response. No significant associations were found with diabetes mellitus, age, angiotensin‐converting enzyme inhibitors use, or baseline creatinine level.

**Conclusions:**

While this study found that BCR‐ABL TKIs can lead to a decline in eGFR, AKI, and CKD, it also demonstrated that they were relatively safer in our study population.

**Abbreviations**
ACEi: angiotensin‐converting enzyme inhibitors
AKI: acute kidney injury
AOR: adjusted odds ratio
ARE: adverse renal event
CHR: complete hematologic response
CI: confidence interval
CKD: chronic kidney disease
CML: chronic myelogenous leukemia
CML‐CP: chronic myeloid leukemia‐chronic phase
CRAE: chronic renal adverse event
DFSP: diffuse fibrosarcoma protuberance
DM: diabetes mellitus
eGFR: estimated glomerular filtration rate
EMR: electronic medical record
GFR: glomerular filtration rate
HTN: hypertension
IQR: interquartile range
MMR: major molecular remission
PDGFR: platelet‐derived growth factor receptor
RFT: renal function test
TASH: Tikur Anbesa Specialized Hospital
TKI: tyrosine kinase inhibitor

## 1 INTRODUCTION

BCR‐ABL tyrosine kinase inhibitors (TKIs) mainly target the BCR‐ABL1 tyrosine kinase but can also target other tyrosine kinases like platelet‐derived growth factor receptor (PDGFR) and KIT. It is used primarily to manage chronic myelogenous leukemia (CML). However, it also has applications in treating BCR‐ABL‐positive acute lymphoblastic leukemia, myelodysplastic syndrome/myeloproliferative neoplasia with PDGFR rearrangement, hyper‐eosinophilic syndrome, and chronic eosinophilic leukemia with FIP1L1‐PDGFRα rearrangement, KIT‐positive gastrointestinal stromal tumor, and diffuse fibrosarcoma protuberance (DFSP) [1, 2].

The use of TKIs has significantly improved outcomes and survival for CML patients [3]. Currently, several TKIs are available, including imatinib, nilotinib, dasatinib, bosutinib, and ponatinib [2]. The choice of TKI for a patient depends on various factors, such as age, disease stage, comorbidities, and individual tolerability. Imatinib is the first‐line therapy for many patients, and it is practical, resulting in primary molecular response (major molecular remission [MMR]) for about 85% of patients in 5 years of treatment. When there is an intolerance or resistance to primary therapy, second‐generation TKIs (dasatinib, nilotinib, and bosutinib) and third‐generation ponatinib can be used [4].

TKIs are more effective and less toxic than conventional chemotherapy [5]. However, understanding the long‐term consequences of TKI exposure is essential, as they are administered for prolonged periods in CML patients [6]. Early complications, which can occur during the initial stages of therapy, include skin rash, fatigue, nausea, myalgia, arthralgia, and edema. Additionally, each TKI may have unique complications associated with its use. Long‐term TKI use can affect various body organ systems, including the cardiovascular, bone marrow, gastrointestinal, liver, and kidney systems. [7, 8].

Kidneys play a role in the pharmacokinetics of TKIs, making them susceptible to their toxicities [9]. However, less attention has been paid to the renal toxicities of TKIs despite several studies showing a possible association between TKI use and renal damage, including glomerular and tubular injuries [5, 6, 10, 11]. The exact mechanism by which TKIs cause renal toxicity remains unknown, but potential mechanisms are discussed below. The first mechanism involves the presence of abundant PDGFR and KIT receptors on podocytes, mesangial cells, and tubular epithelium, which are crucial for their survival and growth. Therefore, inhibiting tyrosine kinase signaling disrupts cellular processes, leading to oxidative stress, inflammation, and apoptosis. Ultimately, this can cause fibrosis, proteinuria, and glomerulosclerosis [12, 13]. Another proposed mechanism involves endothelial damage caused by TKIs, leading to local thrombosis and thrombotic angiopathy that manifests as proteinuria [8]. Additionally, TKIs can inhibit the activity of angiotensin‐converting enzyme (ACE), leading to decreased renal blood flow and glomerular filtration [14].

Studies on the renal toxicities of TKIs have increased in recent years. However, the exact cause of these toxicities remains unknown. Reports suggest that the incidence of acute kidney injury (AKI) in patients taking Imatinib falls between 4 and 7%, while the incidence of CKD is even higher, ranging from 14% to 23% in these patients [5, 6, 11]. The development of these renal issues can negatively impact both medication adherence and overall prognosis. Multiple studies have identified various risk factors associated with TKI‐induced renal toxicities. These include pre‐existing impaired kidney function, the specific type, dose, and duration of the TKI used, age, and other medical conditions like hypertension (HTN) and diabetes mellitus (DM) [5, 6, 9, 11]. Additionally, the use of certain non‐TKI medications (e.g., hydroxyurea and interferon) and specific anti‐hypertensive medications [5].

The extent of TKI‐associated adverse renal events and the factors influencing them remain underexplored in Ethiopia. To our knowledge, this is the first study in the country to address this issue. Given the growing recognition of TKI‐related renal dysfunction, this research aims to elucidate the burden of such toxicities in the Ethiopian patient population.

## 2 MATERIALS AND METHODS

### 2.1 Study setting and design

A hospital‐based cross‐sectional analytic study was conducted from January 2023 to January 2024 at Tikur Anbesa Specialized Hospital (TASH), the only center in the country that provides TKIs to patients. The MAX Foundation donates these TKIs. All the TKIs are freely offered through the generous and continuous support of the Gleevec International Patient Assistance Program. The study included all patients who followed up at the TASH hematology unit, took TKIs, and had at least one documented renal function test (RFT) within the past 3 months to determine the burden and pattern of TKI‐associated renal toxicities. Patients with no documented serum creatinine level at baseline or within the past 3 months were excluded. A total of 260 patients were included in the study.

### 2.2 Study procedure

Data were collected using a structured data collection tool developed by reviewing relevant scientific literature tailored to meet the study objectives. Information on sociodemographic characteristics, comorbidities (HTN, DM, and cardiac disease), laboratory investigations (urine analysis [UAA], serum creatinine, and complete blood count), non‐TKI medications, and treatment outcomes were extracted from the electronic medical records (EMRs).

### 2.3 Sampling procedure

All eligible patients with complete data were involved in the study. Therefore, sample size calculation was not required.

### 2.4 Operational definition

- AKI and CKD were defined as per KIDIGO guidelines.
- TKI‐associated AKI: defined as the same criteria used for general AKI but linked explicitly to TKIs or documentation of AKI in a patient who uses TKI by a physician that was not explained by other causes.
- CKD associated with TKI use defined decrement of eGFR to < 60 mL/min/1.73 m^2^ ≥90 days after initiation of TKI.
- TKI‐associated adverse renal event is defined as a 30% eGFR reduction from baseline or eGFR < 60 mL/min/1.73 m^2^, which occurred for more than 90 days, the development of AKI, or the development of significant proteinuria after the initiation of TKI.
- Significant proteinuria is having more than 500 mg of protein in urine over 24 h.
- Significant proteinuria associated with TKI uses the specific occurrence of significant proteinuria after starting TKI therapy.

### 2.5 Statistical analysis

The data were analyzed using SPSS version 26. Descriptive statistics, including frequencies, percentages, and tables, were used to summarize and present the data. The Wilcoxon Signed‐Ranks test calculated the difference between the baseline eGFR and the most recent eGFR. First, we used a bivariate logistic regression analysis to identify the relationship between the outcome and predictor variables. Multiple logistic regression used variables with a *p*‐value ≤ 0.25 to control confounding factors. The Hosmer‐Lemeshow statistic was not statistically significant (*p* = 0.256), suggesting a good fit for the model. The Nagelkerke R‐squared value is 23.7%, which means the model explains 23.7% of the variation. The analysis confirmed that the independent variables were not highly correlated, as indicated by variance inflation factors below 10. We employed adjusted odds ratios (AORs) with 95% confidence intervals (CIs) to evaluate the relationships between the independent variables and the outcome. We considered an association statistically significant if the *p*‐value was less than 0.05.

## 3 RESULTS

### 3.1 Sociodemographic factors and disease characteristics

A total of 260 patients were included in the study. Among them, 142 (54.6%) were male. The mean age of the patients in the study was 45.15 years (standard deviation ± 14.03). The study population primarily consisted of patients with CML, with 86.5% falling into this category. Notably, 92% of these CML patients were in the chronic phase of the disease, while only a small percentage (4.9% and 3.1%, respectively) were in the accelerated phase or blast crisis.

### 3.2 Baseline hematologic and renal function parameters

The median creatinine level was 0.7 mg/dL (interquartile range [IQR] 0.6–0.8). Similarly, the median eGFR was 115 mL/min/1.73 m^2^ (IQR 101–123). However, only 17 patients had a UAA at baseline. Among those who did, 10 had no abnormalities detected. However, a smaller group (seven patients) exhibited varying levels of proteinuria, ranging from +1 to +3. 90% of patients had GFR stage 1 at diagnosis, followed by 7.7% GFR stage 2 and 2.3% GFR stage 3a. Hematologic and renal function parameters at diagnosis are summarized in Table 1.

**TABLE 1:** Baseline hematologic and renal function parameters at the time of diagnosis.

	Diagnosis	
	CML	Others (DFSP, GIST, and HES)
	Median	Median
WBC (Cells/uL)	229,000	5230
Hemoglobin (g/dL)	10.00	13.00
Platelet (Cells/uL)	349,000	267,000
Baseline creatinine (mg/dL)	0.70	0.70
Baseline eGFR (mL/min/1.73m^2^)	116	103

Abbreviations: CML, chronic myelogenous leukemia; DFSP, diffuse fibrosarcoma protuberance; eGFR, estimated glomerular filtration rate; GIST, gastrointestinal stromal tumor; HES, hyper‐eosinophilic syndrome; WBC, white blood cells.

### 3.3 Comorbidities affecting renal functions

The most common comorbidities were DM and HTN, found in 5.5% and 5.1% of patients. Similarly, the most frequently used non‐TKI medications were CCBs and ACEIs, used in 3.5% and 3.0% of patients, respectively. Table 2 summarizes other comorbidities and medications.

**TABLE 2:** Comorbidities and medications.

	Median	Frequency	Percentage (%)
Total duration of TKI ± IQR (months)	35.15 ± 14.03		
DM		15	5.8%
HTN		14	5.4%
HIV		3	2.3%
Cardiac disease		4	1.5%
Thiazide diuretics		4	1.5%
CCB		9	3.5%
ACEi/ARB		8	3.1%

Abbreviations: ACEI/ARB, angiotensin enzyme inhibitor/angiotensin receptor blocker; CCB, calcium channel blockers; DM, diabetes mellitus; HIV, human immunodeficiency virus; HTN, hypertension; IQR, interquartile range; TKI, tyrosine kinase inhibitor.

### 3.4 Treatment and outcomes

Initially, all patients received imatinib. The daily dosing regimen varied, with 240 patients receiving 400 mg PO, 17 receiving 600 mg PO, and 3 receiving 800 mg PO daily or in divided doses. For CML patients, hydroxyurea was used initially to manage high white blood cell counts and reduce them below 20,000 Cells/uL, followed by the initiation of imatinib. The median duration of treatment was 34.5 (IQR 16–79.5) months. Approximately 19% of patients required a switch to second or third‐line therapy due to resistance or treatment failure. Among these patients, Nilotinib was the most common alternative TKI (57.4%), followed by Bosutinib and Dasatinib (around 18.5% each). A smaller number (5.6%) received Ponatinib.

To assess treatment effectiveness, molecular testing was performed on 104 patients. Notably, 63.5% achieved MMR, defined as *BCR: ABL1* ≤0.1%. However, among all 260 patients, only 63.5 patients achieved either MMR or complete hematologic response (CHR). The remaining 36.5% of patients experienced either hematologic/molecular failure or disease progression.

### 3.5 The magnitude of adverse renal events

TKI treatment led to a statistically significant decrease in eGFR, as measured by the Wilcoxon Signed‐Ranks test (mean difference: −6.365, *p*‐value < 0.001). Adverse renal events (AREs) were defined as any of the following: AKI, significant proteinuria, development of chronic kidney disease (CKD), or a decrease in eGFR by at least 30%. The overall prevalence of AREs was 13.1%. Four patients developed AKI, all of whom recovered but did not receive a nephrologist's follow‐up. Seven patients developed CKD after the initiation of TKI: four with stage 3a, two with stage 3b, and one with stage 4 diseases. Only four of these patients were linked to nephrology follow‐up. Additionally, one patient developed significant proteinuria (2 g/24 h) within the first year of follow‐up. This patient's baseline eGFR was 56 mL/min/1.73 m^2^ and progressed to stage 3b CKD within 50 months. It could be undiagnosed renal disease before treatment initiation. Of the seven patients who developed CKD, only 4 received nephrologist care. Only thirty‐four patients had a recent urinalysis, with results showing protein levels ranging from +1 to +3. 27 patients had protein +1, 4 patients had protein +2, and 3 patients had protein +3. However, only one of these patients underwent a 24‐hour urine protein test. Only four of those patients had AREs. Tables 3 and 4 summarize the characteristics of patients who develop AKI and CKD.

**TABLE 3:** Characteristics of patients who developed acute kidney injury (AKI).

Patient	Age (Year)	Sex	Diagnosis	Baseline eGFR	Last eGFR	Time to AKI development (in months)	Comorbidity	Non‐TKI medications
1	58	Female	CML (AP)	58	74	12	None	None
2	36	Male	CML (CP)	119	73	60	None	None
3	83	Male	GIST	75	60	1	DM and HTN	CCB and Metformin
4	52	Male	CML (CP)	108	91	1	None	None

Abbreviations: AKI, acute kidney injury; AP, accelerated phase; CCB, calcium channel blockers; CML, chronic myelogenous leukemia; CP, chronic phase; DM, diabetes mellitus; eGFR, estimated glomerular filtration rate; GIST, gastrointestinal stromal tumor; HTN, hypertension; TKI, tyrosine kinase inhibitor.

**TABLE 4:** Characteristics of patients who develop chronic kidney disease (CKD).

Patient	Age (Year)	Sex	Diagnosis	Baseline eGFR	Last eGFR	Stage of CKD	Duration of TKI	Comorbidity	Non‐TKI medication	UAA result
1	63	Female	GIST	64	20	4	19	DM and HTN	ACEi/ARBand insulin	Protein +1 24 h –175 mg
2	55	Female	CML (CP)	95	36	3b	169	None	None	NA
3	60	Female	CML (CP)	76	40	3b	86	DM	Metformin	NA
4	50	Female	CML (CP)	111	55	3a	103	None	None	NA
5	61	Female	CML (CP)	77	52	3a	109	HTN	Thiazide and ACEi/ARB	NA
6	71	Male	CML (CP)	73	54	3a	31	None	None	NA
7	66	Female	CML(CP)	62	55	3a	93	None	None	NA

Abbreviations: ACEI/ARB, angiotensin converting enzyme inhibitors/angiotensin receptor blocker; CKD, chronic kidney disease; CML, chronic myelogenous leukemia; eGFR, estimated glomerular filtration rate; GIST, gastrointestinal stromal tumor; NA, not available; TKI, tyrosine kinase inhibitor; UAA, urine analysis.

### 3.6 Factors associated with ARE

Upon binary regression, age, sex, baseline creatinine, total duration of TKI treatment, DM, HIV infection, HTN, use of angiotensin‐converting enzyme inhibitors (ACEi), and treatment outcome were associated with AREs using a p‐value of 0.25 as a cutoff. These variables were then included in a multivariate logistic regression analysis, which revealed that five variables were statistically significantly associated with the development of AREs. These variables were: total duration of TKI treatment (AOR: 1.009, 95% CI: 1.00–1.017, *p* = 0.034), HTN (AOR: 7.651, 95% CI: 1.483–39.479, *p* = 0.015), HIV (AOR: 7.599, 95% CI: 1.184–48.770, *p* = 0.033), male gender (AOR = 0.366, 95% CI = 0.154–0.918, *p* = 0.032), and the combined variable of MMR and CHR (AOR: 2.822, 95% CI: 1.032–7.714, *p* = 0.043). At the same time, the study did not find a statistically significant association between switching to second/third‐line therapy and developing AREs; only four out of 49 patients who switched therapy experienced AREs. Table 5 summarizes these factors associated with AREs.

**TABLE 5:** Factors associated with adverse renal event (ARE).

	ARE					
Variables	Yes	No	COR (95% CI)	*p*‐value	AOR (95% CI)	*p*‐value
Age (mean + SD)			1.043 (1.015–1.07)	0.002	1.0309 (0.997–1.064)	1.03
Sex						
Male	14	128	0.536 (0.258–1.114)	0.095	0.376 (0.154–0.918)	0.032^a^
Female	20	98				
Baseline creatinine (mean)	0.78	0.72	4.722 (0.859–25.947)	0.074	5.45 (0.577–51.495)	0.139
Total duration of TKI treatment in months (mean)	66.5	45.9	1.009 (1.002–1.016)	0.014	1.009 (1.00–1.017)	0.034^a^
DM						
Yes	4	11	0.384 (0.115–1.282)	0.12	0.708 (0.170–2.951)	0.636
No	30	215				
HTN						
Yes	7	7	8.111 (2.643– 24.892)	<0.0001	7.651 (1.483–39.479)	0.015^a^
No	27	219				
HIV						
Yes	2	4	3.469 (0.61–19.711)	0.161	7.599 (1.184–48.77)	0.033^a^
No	32	222				
Use of ACEi						
Yes	4	4	7.4 (1.756–31.152)	0.006	1.519 (0.17– 13.592)	0.709
No	30	222				
Treatment outcome						
MMR and CHR	26	139	2.034 (0.881–4.695)	0.096	2.822 (1.032–7.714)	0.043^a^
Not in MMR and CHR	8	87				

Abbreviations: ACEI, angiotensin‐converting enzyme inhibitor; AOR, adjusted odds ratio; ARE, adverse renal event; CHR, complete hematologic response; CI, confidence interval; COR, crude odds ratio; DM, diabetes mellitus; HIV, human immunodeficiency virus; HTN, hypertension; MMR, major molecular remission; SD, standard deviation.

^a^ Statistically significant.

## 4 DISCUSSION

This study assessed the effect of BCR‐ABL TKIs associated with renal toxicities and associated factors in patients taking those medications for various reasons. The gender distribution showed slightly male predominant, possibly because CML is more common in men [15]. Although a previous study found an association between the male sex and the development of chronic renal injury in 564 patients with CML‐CP [5], our study found that the male sex had a protective effect, with an AOR of 0.376 (95% CI: 0.154–0.918; *p*‐value = 0.032). One study reported an association between renal toxicities and female sex, which supports our findings [16].

We found a statistically significant decline in eGFR of 6.365 mL/min/1.73 m^2^ over a median follow‐up of 34.5 months in patients receiving Imatinib treatment. This finding aligns with previous studies. Yilmaz et al. reported a steeper initial decline in their study, with a mean decrease of 8 and 10 mL/min/1.73 m^2^ at 3 and 6 months, respectively, before stabilizing after four years. Conversely, dasatinib showed a smaller initial decline (3 and 4 mL/min/1.73 m^2^ at 3 and 6 months) and potentially stabilized after one year. Interestingly, Nilotinib increased eGFR in a similar study, highlighting the varying effects of different TKIs on kidney function [6]. Marcolino et al. observed a significant decrease in eGFR with longer Imatinib treatment duration (average 2.77 mL/min/1.73 m^2^ per year) [11]. Additionally, a meta‐analysis of nine studies revealed a significant eGFR decline in patients taking Imatinib in six of the studies [17].

In our study, the incidence of AKI was 1.5%, lower than the reported rates in other studies (Molica et al. and Yilmaz et al.: 4%, Marcolino et al.: 7%) [6, 9, 11]. One possible explanation for this disparity could be the unique characteristics of our study population. Our patients, with a median age of 45 years and a lower comorbidity burden, differ significantly from those in the Molica et al. (median age: 57.5 years) and Yilmaz et al. (median age: 48 years at the initiation of TKI, followed for 52 months) studies. Furthermore, the prevalence of DM, HTN, and CKD in our patients was only 5.8%, 5.4%, and 1.5%, respectively, compared to the Yilmaz et al. study's baseline prevalence of 25% hypertension, 7% diabetes mellitus, 9% coronary artery disease, and 11% CKD [6]. Similarly, Marcolino et al. found a prevalence of 28% hypertension, 7% diabetes mellitus, and 5% CKD [11]. These findings may explain the low incidence of AKI in our patients. Importantly, all AKI cases in our study were resolved without requiring discontinuation of the TKI therapy. This contrasts with Marcolino's study, where only 1 out of 7 patients with AKI recovered. In our research, the resolution of AKI was achieved with only supportive care [11].

The prevalence of CKD was substantially lower in our study (2.7%) compared to 23% in Xin et al.’s imatinib group [5]. Similarly, our study observed a significantly lower incidence of AREs at 13.1% compared to the 44% prevalence of chronic renal adverse events (CRAEs) reported by Xin et al. in their imatinib cohort. The study established CRAE based on two criteria: a reduction in eGFR by 30% compared to baseline or a sustained eGFR below 60 mL/min/1.73 m^2^ for a minimum duration of 90 days. Several factors may explain these differences. First, Xin et al.’s study population had a higher baseline burden of kidney‐related comorbidities (16% vs. 11.2% in our study), including hypertension and diabetes. Secondly, a more significant proportion of patients in their research (20%) were using medications like antihypertensives and diabetes treatments, which can potentially affect kidney function. Additionally, the median baseline eGFR was significantly lower in Xin et al.’s study (94 vs. 115 in our study), indicating a potentially more compromised kidney function at baseline in their cohort [5]. These findings are consistent with other studies reporting a higher incidence of CKD in patients treated with imatinib. For instance, one study observed a 14% CKD rate among 468 newly diagnosed CML‐CP patients receiving TKIs, with 82% taking imatinib [6]. Similarly, another study found CKD in 16% of 105 patients on imatinib therapy [11].

The duration of TKI treatment was found to have a statistically significant association with ARE with an AOR of 1.001 (95% CI: 1–1.017, *p*‐value: 0.034). Similar associations were found in several studies [6, 18, 19]. Our study found a significant association between MMR CHR and AREs (AOR = 2.822, CI: 1.032–7.714, *p*‐value: 0.043). On the contrary, a study by Yimaz et al. found an association but not a significant association with AKI or CKD [6].

HTN was associated with the development of ARE with AOR (7.651; 95% CI, 1.483–39.479; *p* = 0.015). It is consistently demonstrated in various studies [6, 11]. A previous retrospective study analyzed 142 patients with CML and found a rapid decline in eGFR in patients taking ACEi compared to those not taking it. In contrast, our study did not identify a significant association between ACEi use and AREs. This difference may be attributable to our research's limited sample size of ACEi patients [20].

Our study found that HIV is significantly associated with ARE with an AOR of 7.599 (95% CI: 1.184–48.770, *p* = 0.033). No other studies have reported this association. Diabetes was found to be associated with the development of AREs [6, 9, 18]. However, our study did not find a strong association.

While age was an independent risk factor for developing TKI‐associated renal toxicities in different literature [5, 6, 9, 11, 13, 18]. Our study did not identify a significant association between these factors. Using a standard dose of imatinib or a decreased dose did not result in a substantial difference in mean eGFR [18]. We also observed similar findings with no difference between patients receiving various doses of imatinib 400–800 mg daily.

Use of previous non‐TKI treatment (such as hydroxyurea, interferon, and chemotherapy) was found to be associated with TKI‐associated renal toxicities [5]. In our case, almost all patients received hydroxyurea, and it was difficult to assess the effect.

There is mixed evidence regarding whether changing imatinib to second or third‐line therapy or discontinuing imatinib improves renal dysfunction. One study found that changing imatinib to second‐line therapy improves renal dysfunction, even in patients with new‐onset CKD [13]. Another study showed no significant difference in eGFR after TKI discontinuation in patients in treatment‐free remission or molecular relapse [19]. In our case, there is no increased risk in patients taking imatinib compared to those switched to second or third‐line therapy.

### 4.1 Strengths and limitations of the study

This study, despite its limitations, offers valuable insights. It is the first African study to investigate the relationship between BCR‐ABL TKIs and renal toxicities, and it does so with a thorough and structured approach, examining numerous factors that could influence adverse renal events. However, the cross‐sectional design hinders establishing cause‐and‐effect, and the sample size might limit generalizability. Additionally, limited data on baseline proteinuria and the lack of periodic measurements of RFTs are shortcomings. We recommend replicating this study in a large sample size and multicenter prospective cohort. We also recommend conducting this study on patients without additional comorbidities.

## 5 CONCLUSION

This study found that treatment with BCR‐ABL TKIs decreased eGFR, but the rate of severe adverse kidney events was lower than reported in some other studies. This could be due to the difference in the patient population, which was younger and had fewer pre‐existing comorbidities. The study also identified factors associated with a higher risk of adverse events, such as longer treatment duration, hypertension, and HIV infection. Interestingly, achieving major molecular remission or CHR was also linked to a higher risk. These findings suggest that TKIs are relatively safe for Ethiopia patients. However, it is crucial to emphasize that further research with larger and more diverse populations is needed to confirm these findings and guide clinical practice.

## AUTHOR CONTRIBUTIONS

**Zekarias Seifu Ayalew**: Conceptualization; methodology; investigation; analysis and writing of the manuscript. **Gebeyehu Tessema Azibte**: Conceptualization; methodology; investigation; analysis and writing of the manuscript. **Fisihatsion Tadesse**: Conceptualization; methodology; investigation; analysis and writing of the manuscript. **Biruk Abate Legesse**: Methodology; data curation; drafting; interpretation and edition of the data; supervision and edition of the manuscript. **Zerubabel Getahun Kiflu**: Methodology; data curation; drafting; interpretation and edition of the data; supervision and edition of the manuscript. **Mahlet Tsige Weldeamanuel**: Methodology; data curation; drafting; interpretation and edition of the data; supervision and edition of the manuscript. **Kibrekidusan Aynekulu Tsige**: Methodology; data curation; drafting; interpretation and edition of the data; supervision and edition of the manuscript. **Bereket Abraha Molla**: Methodology; data curation; drafting; interpretation and edition of the data; supervision and edition of the manuscript. **Addisu Melkie Ejigu**: Conceptualization; methodology; investigation; analysis and writing of the manuscript.

## CONFLICT OF INTEREST STATEMENT

The authors declare no conflict of interest.

## FUNDING INFORMATION

The authors received no specific funding for this work.

## ETHICS STATEMENT

The study was conducted by the Declaration of Helsinki and approved by the Institutional Review Board of Addis Ababa University, College of Medicine and Health Sciences protocol.

## PATIENT CONSENT STATEMENT

Informed consent was obtained from all subjects involved in the study.

## CLINICAL TRIAL REGISTRATION

The authors have confirmed clinical trial registration is not needed for this submission.

## Supporting information



Supporting information

## Data Availability

The authors confirm that the data supporting the findings of this study are available in the article.
