# Supplementary material for: Unveiling the nephrotoxic profile of BCR‐ABL tyrosine kinase inhibitors: A real‐world experience in Africa
Source: EJHaem. 2024 Jul 31;5(4):749–56. doi: 10.1002/jha2.988 (PMC11327723; doi:10.1002/jha2.988)
Supplement: Supplementary file 1 — Supporting information [file JHA2-5-749-s001.docx]

**Tweetable Summary Label**

Please supply a tweet (up to 250 characters) for possible use by the social media editor on the Publisher’s Twitter account if your paper is accepted.

BCR-ABL tyrosine kinase inhibitors are safe for kidney function in African chronic myeloid leukemia patients, with a manageable decline over time. However, certain factors may increase risk. #Ethiopia #Healthcare

**Twitter Handle(s) (up to 7 authors)**

Please include up to seven Twitter handles that may be appropriate to mention in conjunction with your tweet.

Zack_MD1

GebeyehuTessema

LinkedIn Accounts

https://www.linkedin.com/in/zekarias-md

https://www.linkedin.com/in/gebeyehu-tessema-2904471aa
